# Prenatal diagnosis and long‐term follow‐up of a Chinese patient with mosaic variegated aneuploidy and its molecular analysis

**DOI:** 10.1002/ccr3.2802

**Published:** 2020-05-19

**Authors:** Sheng Mou Lin, Ho Ming Luk, Ivan Fai Man Lo, Wai‐Keung Tam, Kelvin Yuen Kwong Chan, Hei‐Yee Tse, Wing Cheong Leung, Mary Hoi Yin Tang, Anita Sik Yau Kan

**Affiliations:** ^1^ Department of Obstetrics and Gynaecology The University of Hong Kong‐Shenzhen Hospital Shenzhen China; ^2^ Department of Health Clinical Genetic Service Hong Kong Hong Kong; ^3^ Department of Obstetrics and Gynaecology Queen Mary Hospital Hong Kong Hong Kong; ^4^ Prenatal Diagnostic Laboratory Tsan Yuk Hospital Hong Kong Hong Kong; ^5^ Department of Obstetrics and Gynaecology Kwong Wah Hospital Hong Kong Hong Kong; ^6^ Department of Obstetrics and Gynaecology The University of Hong Kong Hong Kong Hong Kong

**Keywords:** molecular diagnosis, mosaic variegated aneuploidy, prenatal diagnosis

## Abstract

Mosaic variegated aneuploidy (MVA) is a rare genetic disorder caused by mutations in *BUB1B*, *CEP57,* or *TRIP13*. We describe the prenatal diagnosis, molecular characterization, and clinical management of a long‐lived patient with *BUB1B*‐related MVA.

## Graphical abstract

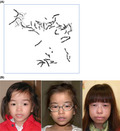

## 1 INTRODUCTION

Mosaic variegated aneuploidy (MVA, OMIM 257300) is a congenital autosomal recessive disorder characterized by mosaic aneuploidies, predominantly trisomies, and monosomies, involving multiple chromosomes and tissues.^1^ Mutations in *BUB1B*, *CEP57,* and *TRIP13* genes, which are involved in mitotic spindle and microtubule stabilization, are responsible for the molecular pathogenesis of MVA. The clinical features of MVA syndrome include severe pre/postnatal growth retardation, microcephaly, central nervous system anomalies, intellectual disability, minor congenital malformation, and predisposition to malignancy. There is some genotype‐phenotype correlation (Table 1). Intellectual disability, microcephaly, brain malformations, epilepsy, and cancer predisposition are more common in *BUB1B* subtype. Rhizomelic shortening of the upper limbs, skull anomalies with conserved head circumference, and absence of malignancy are more common in *CEP57* subtype. In addition, *TRIP13* subtype has growth retardation with microcephaly and developmental delay, but there is no other structural abnormality and dysmorphic facial feature as in *BUB1B* subtype.

**Table 1:** Subtype of Mosaic Variegated Aneuploidy syndrome and genotype‐phenotype correlation

Title	Mosaic variegated aneuploidy syndrome 3; MVA3	Mosaic variegated aneuploidy syndrome 1; MVA1	Mosaic variegated aneuploidy syndrome 2; MVA2	Our case
Inheritance	Autosomal recessive	Autosomal recessive	Autosomal recessive	Autosomal recessive
Molecular basis	Caused by mutation in the thyroid hormone receptor interactor 13 gene (*TRIP13*)	Caused by mutation in the budding uninhibited by benzimidazoles 1 beta gene (*BUB1B*)	Caused by mutation in the 57‐kD centrosomal protein gene (*CEP57*)	Caused by mutation in the budding uninhibited by benzimidazoles 1 beta gene (*BUB1B*)
Laboratory abnormalities	Aneuploidy Premature chromatid separation Chromosome instability	Mitotic lymphocyte and fibroblast cultures show mosaic variegated aneuploidy More than 50% of mitotic cells show premature chromatid separation (PCS) affecting all chromosomes Anaphase loss or nondisjunction with trisomies, tetrasomies, monosomies	Mitotic lymphocyte and fibroblast cultures show mosaic variegated aneuploidy (50%) affecting all chromosomes Chromosomal structural abnormalities	Mitotic lymphocyte and fibroblast cultures show mosaic variegated aneuploidy More than 50% of mitotic cells show premature chromatid separation (PCS) affecting all chromosomes Anaphase loss or nondisjunction with trisomies, tetrasomies, and monosomies
Growth	Height Short stature Other Growth retardation	Height Short stature Weight Low birthweight Low postnatal weight Other Growth retardation, prenatal and postnatal	Height Short stature Weight Intrauterine growth retardation (IUGR) Low birthweight Other Poor growth	Height Short stature Weight Low birthweight Low postnatal weight Other Growth retardation, prenatal and postnatal
Head & neck	Head Microcephaly (in some patients)	Head Microcephaly, severe Brachycephaly Face High forehead Midface hypoplasia Micrognathia Long philtrum Ears Low‐set ears Posteriorly rotated ears Eyes Hypertelorism Upslanting palpebral fissures Epicanthal folds Cataracts Nystagmus Nose Short, wide nose Depressed nasal bridge Anteverted nares Mouth Cleft palate Triangular shaped mouth Neck Short neck	Head Microcephaly Temporal bossing (in 1/4 patients) Frontal bossing (in 1/4 patients) Narrow head (in 1/4 patients) Dolichocephaly (in 2/4 patients) Face Long face (in 1/4 patients) Triangular face (in 2/4 patients) Micrognathia (in 2/4 patients) Ears Hearing impairment (in 1/4 patients) Low‐set ears (in 2/4 patients) Eyes Deep‐set eyes (in 1/4 patients) Short palpebral fissures (in 1/4 patients) Downslanting palpebral fissures (in 2/4 patients) Epicanthal folds (3/4 patients) Nose Small nose (in 2/4 patients) Flat nasal bridge (in 2/4 patients)	Head Microcephaly, severe Face High forehead Midface hypoplasia Long philtrum Ears Low‐set ears Eyes Hypertelorism Epicanthal folds Nose Short, wide nose Depressed nasal bridge Neck Short neck
Cardiovascular			Heart Congenital heart defects (in 2/4 patients) Atrial septal defect Ventricular septal defect Aortic valve regurgitation Vascular Aortic coarctation Subaortic stenosis	Heart Congenital heart defects Pericardial effusion
Respiratory			Lung Abnormal lung lobation (in 1/4 patients)	
Chest		Ribs Sternum Clavicles and Scapulae Short sternum		pleural effusion, chylothorax
Abdomen		Gastrointestinal Feeding difficulties	Gastrointestinal Duodenal atresia (in 1/4 patients)	Gastrointestinal ascites duplication cyst of gut
Genitourinary		Ambiguous genitalia External Genitalia (Male) Micropenis Hypospadias Bifid scrotum Internal Genitalia (Male) Cryptorchidism Kidneys Renal cysts Wilms tumor		Kidneys Wilms tumor glomerulosclerosis
Skeletal			Delayed bone age (in 1/4 patients) Skull Epidermoid cysts (in 1/4 patients) Limbs Rhizomelic shortening of the upper limbs (in 2/4 patients) Hands Clinodactyly (in 2/4 patients)	
SKIN, nails, & hair	Skin Pigmentary abnormalities		Skin Cafe‐au‐lait spot (in 1/4 patients)	Skin Cafe‐au‐lait spot
Neurologic	Central Nervous System Developmental delay (in some patients) Seizures (in some patients)	Central Nervous System Developmental delay, profound Mental retardation, profound Hypotonia, generalized Seizures, generalized tonic‐clonic Seizures, myoclonic Hypoplastic cerebrum Pachymacrogyria Cerebral oligogyria Hypodysplasia of the corpus callosum Agenesis of the corpus callosum Posterior fossa malformations Dandy‐Walker malformation Enlarged ventricles Hydrocephalus Cerebellar hypoplasia	Central Nervous System Mild mental retardation (in 1/4 patients) Hypotonia (in 1/4 patients) Sleep apnea (in 1/4 patients)	Central Nervous System Developmental delay, profound Mental retardation, profound
Endocrine features			Growth hormone deficiency (in 1/4 patients) Hypothyroidism (in 2/4 patients)	
Immunology		Combined immunodeficiency (reported in 1 patient)		
Neoplasia	Wilms tumor	Propensity to tumor development Wilms tumor Nephroblastoma Rhabdomyosarcoma Leukemia		Propensity to tumor development Wilms tumor Nephroblastoma Sertoli‐Leydig cell tumor
Prenatal manifestations		Amniotic Fluid Oligohydramnios Delivery Premature labor		Biochemical serum screening raised maternal serum alpha fetoprotein
Miscellaneous	Onset of Wilms tumor in early childhood Highly variable phenotype other than Wilms tumor	Variable phenotype Heterozygous parents are phenotypically normal but their cells show premature chromatid separation trait (PCS, OMIM 176430)	Four patients have been reported (as of July 2011) Highly variable phenotype Facial dysmorphic features are mild	

Note

Modified and updated from: https://omim.org/clinicalSynopsis/table?mimNumber=617598,614114,257300

Several cases of MVA were diagnosed in prenatal period,^2^ followed by the termination of pregnancy. Here we reported a Chinese patient with the longest survival in literature, with cytogenetic and antenatal findings together with her long‐term postnatal course and molecular finding.

## 2 CASE REPORT

**Figure 1:**
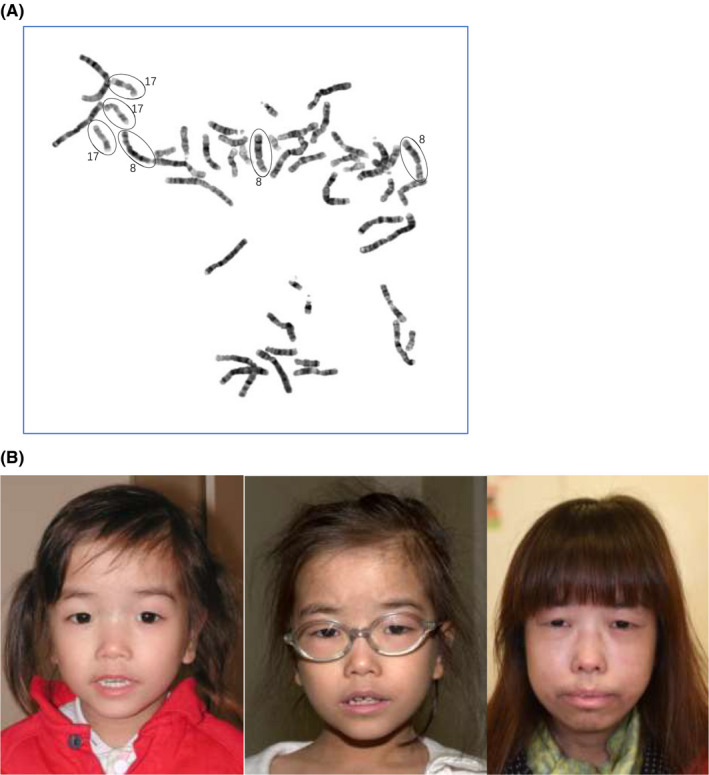
Chromosome study and facial feature of the MVA patient. A, One representative cultured amniocyte metaphase image showing karyotype result of 48,XX,+8,+17. B, Subtle facial dysmorphism at 4, 8, and 22 years old: The patient has microcephaly, failure to thrive with all growth parameters less than 3rd percentile, and subtle dysmorphism (hypertelorism, high forehead, epicanthic fold, and midface hypoplasia). The hypertrichosis at 22 years old was due to side effect of cyclosporine A after renal transplantation

A 29‐year‐old multipara Chinese woman with two previous normal deliveries was referred for invasive prenatal diagnosis at 19 weeks of gestation in 1996. On antenatal screening, she was noted to have raised maternal serum alpha fetoprotein level of 3.08 MoM, abnormal fetal scan with early onset growth retardation, pericardial effusion and ascites, congenital heart disease, and duplication cyst of gut. Chromosome study of cultured amniotic fluid cells showed multiple cell line with a composite karyotype of 45~51,XX,+X[1],+2[3],+3[2],+5[6],‐5[1],+6[4],−6[2],+7[6],+8[3],+10[3],+12[1],+14[1],+15[1],+16[1],+17[7],+18[1],+20[1],+21[2][cp150] (Figure 1A). After counseling, the couple opted for keeping the fetus and the baby was delivered at 37 weeks with birthweight of 1.55 kg (<−3.6SD) and body length of 45 cm (<−3.2SD). Chromosome study of placental tissue showed a composite karyotype as 45~51,XX,−X[2],+1p[2],+1q[1],−1[2],+2[3],−2[3],+5[2],−5[3],+7[4],+8[9],+9[2],−10[2],+11[5],+12[4],+13[1],+14[1],‐16[1],+17[3],+18[3],‐18[1],+19[4],+20[5],+21[2],+fra[1][cp44]. Chromosome study of cord blood lymphocytes showed 45~47,XX,−X[1],−13[1],+18[1],−21[1][cp8].

**Figure 2:**
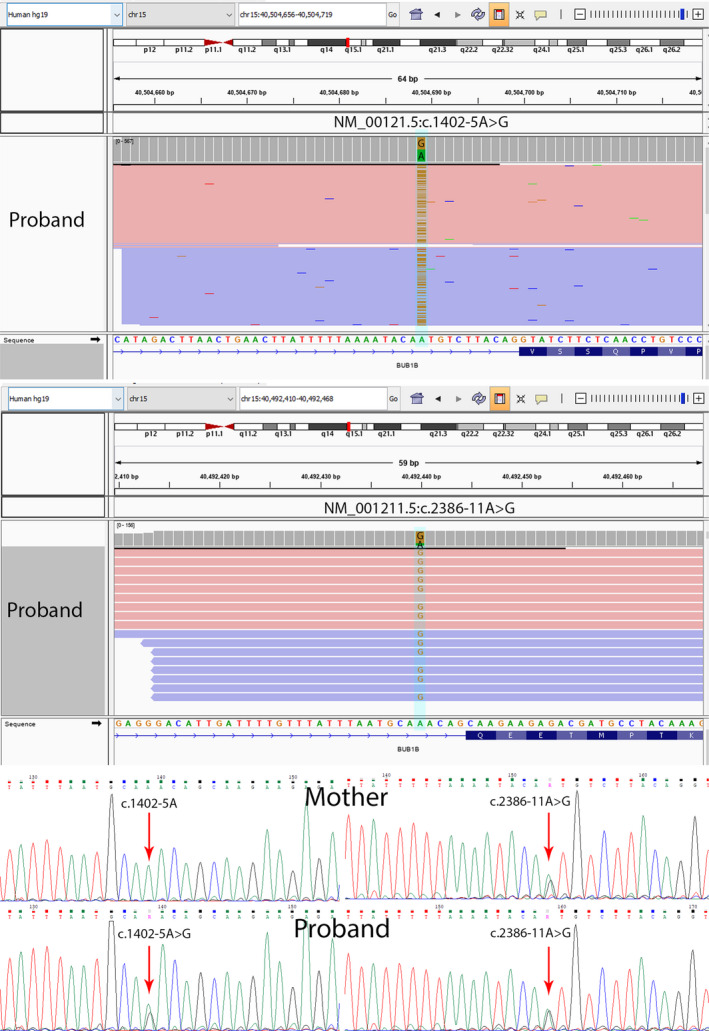
Integrated genomic viewer to show the compound heterozygous pathogenic variants of *BUB1B* in patient. (Upper panel showed there is change from wild‐type variant A to mutant type variant G at nucleotide position c.1402‐5, while the middle panel showed there is a change from wild‐type variant A to mutant type variant G at nucleotide position c.2386‐11. The lower electropherogram confirmed the mutations by Sanger sequencing. Reference sequence NM_001211.5(BUB1B)

Based on the cytogenetic findings and clinical features, the clinical diagnosis of mosaic variegated aneuploidy syndrome was made. The proband was then regularly followed in pediatric and genetic department. At 8 months of age, she was diagnosed to have infantile neuroblastoma with surgical excision. She had failure to thrive and microcephaly all along. Developmental assessment at 2 years of age showed mild global developmental delay. She also had subtle facial dysmorphism (hypertelorism, high forehead, epicanthic fold, and midface hypoplasia) on physical examination (Figure 1B). At 15 years old, she was diagnosed to have chronic glomerulosclerosis complicated with chronic renal failure that required renal transplant at 20 years old. At age 21, she developed pleural effusion, chylothorax, bilateral ovarian tumor (Meigs syndrome) with histological confirmation as Sertoli‐Leydig cell tumor. Exome‐sequencing analysis was performed on the DNA extracted from the peripheral blood of the patient. Compound heterozygous variants c.1402‐5A>G and c.2386‐11A>G in *BUB1B* gene were found (Figure 2). Mother is a heterozygous c.2386‐11A>G carrier while father is not available for testing.

## 3 DISCUSSION

Premature chromatid separation (PCS) and asynchrony of mitotic stages is described to be the pathogenic mechanism for mosaic variegated aneuploidies (MVA). PCS/MVA manifests cytogenetically as a variety of mosaic aneuploidies, especially trisomies, double trisomies, and monosomies. The proportion of aneuploid cells varies but is usually >25% and is substantially greater than in normal individuals. Conventional cytogenetic analysis with at least two independent amniocyte cultures should always be performed to diagnose prenatal MVA. In our case, amniocyte, placenta, and cord blood lymphocytes culture demonstrated that the proportion of aneuploidy cells was more than 25%, which confirmed the prenatal diagnosis of MVA.

The phenotype is highly variable across individuals of MVA. Common abnormalities of MVA include intrauterine growth retardation, microcephaly, dysmorphic features, and mental retardation. There is also a high risk of early‐onset childhood cancer like Wilms tumor, rhabdomyosarcoma, or leukemia. Facial dysmorphic features in MVA syndrome include micrognathia, frontal bossing, triangular face, epicanthic folds, hypertelorism, low‐set ears, and broad nasal bridge. Cardiovascular, neurological, skeletal anomalies like rhizomelic shortening of the upper limbs, gastrointestinal, and dermatological anomalies, immunodeficiency, and endocrine problem like hypothyroidism have also been described.^3^ Microcephaly was most commonly observed in MVA cases, described in general 90% patients. Prenatal ultrasound findings in association with MVA included intrauterine growth restriction, microcephaly, Dandy‐Walker malformation, cerebral ventricular dilatation, fetal ascites, oligohydramnios, and increased nuchal translucency. According the literature on the prenatal cases of MVA, fetal growth restriction is the commonest feature. In this case, the main prenatal sonographic features include fetal growth restriction, microcephaly, pericardial effusion, ascites, and congenital heart disease. In the long‐term follow‐up of this patient, she manifested with failure to thrive, microcephaly, mild intellectual disability, and cancer predisposition. As the clinical phenotype is highly heterogeneous in MVA, especially in prenatal period, MVA syndrome is usually under‐recognized and missed.

Genetic defect of chromosome segregation in cell mitosis might be associated with MVA, and mutations of *BUB1B* involved in the mitotic spindle checkpoint might underlie MVA. The compound heterozygous variants NM_001211.5(BUB1B):c.[1402‐5A>G];[2387‐11A>G] found in this patient are located at the RNA splicing acceptor site of exons 11 and 19 of *BUB1B*, and reported in the literature.^4^, ^5^ Reduced *BUB1B* expression on the spindle checkpoint is dose‐dependent. An analogous mutation (in mice) to the human MVA *BUB1B* (encodes protein BUBR1) has a reduced lifespan and develop several age‐related phenotypes at an accelerated rate. Sustained high expression of BUBR1 preserves genomic integrity and reduces tumorigenesis by correcting mitotic checkpoint impairment and microtubule‐kinetochore attachment defects.^6^

Clinical management of patients with MVA syndrome includes symptomatic support and tumor surveillance, particularly for *BUB1B* subtype. Early molecular diagnosis might enable risk stratification for tumor surveillance. Common tumors reported in *BUB1B*‐associated MVA syndrome are Wilms tumor, rhabdomyosarcoma, leukemia and granulosa cell tumor of the ovary. In this case, patient suffered from infantile neuroblastoma and Sertoli‐Leydig cell tumor. It is recommended that patients with MVA syndrome have regular abdominal ultrasound surveillance, to particularly look for Wilms tumor. However, as the incidence rate of other rare tumor in MVA is unknown, there is still no evidence to indicate that routine screening is beneficial. But high index of suspicion is necessary. In case there are any clinical symptoms of malignancy, further investigation should be carried out.

We have reported the longest Chinese survivor of *BUB1B*‐related MVA syndrome in the literature, its clinical course, and management.

## CONFLICT OF INTEREST

There is no any conflict of interest in relation to the work.

## AUTHOR CONTRIBUTIONS

Sheng Mou Lin and Ho Ming Luk: involved in conceptualization, methodology, drafting, and writing manuscript. Ivan Fai Man Lo: involved in data collection and curation. Wai‐Keung Tam and Kelvin Yuen Kwong Chan: carried out investigation. Hei‐Yee Tse, Wing Cheong Leung, and Mary Hoi Yin Tang: involved in data curation and validation. Anita Sik Yau Kan: involved in conceptualization, reviewing, and editing.

## INFORMED CONSENT

Written informed consent was obtained from the patient.
